# Deciphering hub genes and immune landscapes related to neutrophil extracellular traps in rheumatoid arthritis: insights from integrated bioinformatics analyses and experiments

**DOI:** 10.3389/fimmu.2024.1521634

**Published:** 2025-01-08

**Authors:** Yang Li, Jian Liu, Yue Sun, Yuedi Hu, Qiao Zhou, Chengzhi Cong, Yiming Chen

**Affiliations:** ^1^ Department of Rheumatology, The First Affiliated Hospital of Anhui University of Chinese Medicine, Hefei, Anhui, China; ^2^ First Clinical Medical School, Anhui University of Chinese Medicine, Hefei, Anhui, China; ^3^ Institute of Rheumatology, Anhui Academy of Chinese Medicine, Hefei, Anhui, China

**Keywords:** rheumatoid arthritis, neutrophil extracellular traps, neutrophils, synovial microenvironment, inflammation, systems bioinformatics

## Abstract

**Background:**

Rheumatoid arthritis (RA) is a chronic autoimmune disease characterized by synovial inflammation and progressive joint destruction. Neutrophil extracellular traps (NETs), a microreticular structure formed after neutrophil death, have recently been implicated in RA pathogenesis and pathological mechanisms. However, the underlying molecular mechanisms and key genes involved in NET formation in RA remain largely unknown.

**Methods:**

We obtained single-cell RNA sequencing data of synovial tissues from the Gene Expression Omnibus (GEO) database and performed cellular annotation and intercellular communication analyses. Subsequently, three microarray datasets were collected for a training cohort and correlated with a bulk RNA-seq dataset associated with NETs. Differentially expressed genes were identified, and weighted gene correlation network analysis was used to characterize gene association. Using three machine learning techniques, we identified the most important hub genes to develop and evaluate a nomogram diagnostic model. CIBERSORT was used to elucidate the relationship between hub genes and immune cells. An external validation dataset was used to verify pivotal gene expression and to construct co-regulatory networks using the NetworkAnalyst platform. We further investigated hub gene expression using immunohistochemistry (IHC) in an adjuvant-induced arthritis rat model and real-time quantitative polymerase chain reaction (RT-qPCR) in a clinical cohort.

**Results:**

Seven cellular subpopulations were identified through downscaling and clustering, with neutrophils likely the most crucial cell clusters in RA. Intercellular communication analysis highlighted the network between neutrophils and fibroblasts. In this context, 4 key hub genes (CRYBG1, RMM2, MMP1, and SLC19A2) associated with NETs were identified. A nomogram model with a diagnostic value was developed and evaluated. Immune cell infiltration analysis indicated associations between the hub genes and the immune landscape in NETs and RA. IHC and RT-qPCR findings showed high expression of CRYBG1, RMM2, and MMP1 in synovial and neutrophilic cells, with lower expression of SLC19A2. Correlation analysis further emphasized close associations between hub genes and laboratory markers in patients with RA.

**Conclusion:**

This study first elucidated neutrophil heterogeneity in the RA synovial microenvironment and mechanisms of communication with fibroblasts. CRYBG1, RMM2, MMP1, and SLC19A2 were identified and validated as potential NET-associated biomarkers, offering insights for diagnostic tools and immunotherapeutic strategies in RA.

## 1 Introduction

Rheumatoid arthritis (RA) is a common chronic autoimmune disease characterized by persistent synovial hyperplasia, inflammation, and gradual joint damage (1). Patients with RA usually present with symmetric polyarticular pain primarily affecting the small joints of the hands and feet. However, as the disease progresses, it may involve bone destruction in the larger joints and damage to other organs (2). RA affects approximately 0.5% to 1.0% of the global population and heavily burdens patients’ quality of life due to its disabling nature, multisystemic damage, and increased mortality (3, 4). Although substantial progress has been made in RA management and research, the exact mechanisms underlying its pathogenesis remain poorly understood, and effective tools to identify patients at risk, aid treatment, and predict prognosis are still lacking. Thus, a thorough understanding of the cellular features and molecular mechanisms within the synovial microenvironment in RA and identifying new molecular biomarkers are essential to improving clinical diagnosis and treatment.

It is widely recognized that RA pathogenesis is a complex process involving multiple interacting factors, including genetics, environmental factors, immune dysfunction, and metabolic and infectious factors (5, 6). Synovial tissue is the primary site of the RA inflammatory cascade and exhibits remarkable diversity and heterogeneity. Tissue-resident mesenchymal cells, known as fibroblast-like synoviocytes (FLS), engage in a complex dialogue and response with infiltrating immune cells, leading to qualitative changes in the cellular phenotype, resulting in an intricate inflammatory microenvironment (7). Recent accumulating evidence reveals the important role of neutrophil-dominated innate immunity in the pathogenesis of RA (8). Following bacterial or viral stimulation, numerous neutrophils are activated and recruited to inflamed synovial tissues and joints, generating various effector mechanisms, including phagocytosis, degranulation, delayed apoptosis, and the release of reactive oxygen species (9). Notably, most of the existing studies, which have focused on FLS-centered phenotypic identification and its mediated immune response, have largely overlooked the critical role of neutrophils (10). Moreover, the pathway mechanisms of how neutrophils interact with other microenvironmental components, especially FLS, are still poorly understood. Therefore, additional investigations are essential to enhance our mechanistic understanding of RA and the development of effective intervention pathways.

Among the effector functions of neutrophils, an ultramicroscopic meshwork of DNA and various cytoplasmic and granule proteins, known as neutrophil extracellular traps (NETs), has been a focus of neutrophil research (11). NETs often play complex, dual roles in various autoimmune diseases, inflammation and cancer (12, 13). Initially, NETs were proposed as a defense mechanism that traps and kills pathogens, thereby protecting the host from aggression (14). Recent studies indicate that NETs can activate FLS, which may serve as key drivers in initiating and perpetuating RA’s inflammatory pathology (15). Elevated biomarkers demonstrate considerable diagnostic value in RA, suggesting their potential utility for identifying individuals at risk and initiating prophylactic treatments (16, 17). Similarly, multiple experimental studies have demonstrated the feasibility of targeting NET formation in RA therapeutic strategies. For example, Zhao et al. reported a study on MSC infusion therapy to ameliorate inflammatory arthritis by inhibiting NETs formation through the PGE2-PKA-ERK signaling pathway (18). Shu et al. found that populin inhibited the entry of MPO and PADI4 into the nucleus to block the release of NETs, thereby ameliorating RA inflammation (19). Recently, a single-cell mapping analysis revealed the heterogeneity of neutrophils in periodontitis tissues and constructed a predictive model of key genes associated with NETs by a machine learning algorithm (20). However, studies on neutrophil function and hub genes associated with NETs involved in the synovial microenvironment of RA remain sparse. In the context of existing studies, identifying NET-related genetic biomarkers will enhance our understanding of these mechanisms and provide more potential targeted therapeutic strategies for RA.

**Figure 1:**
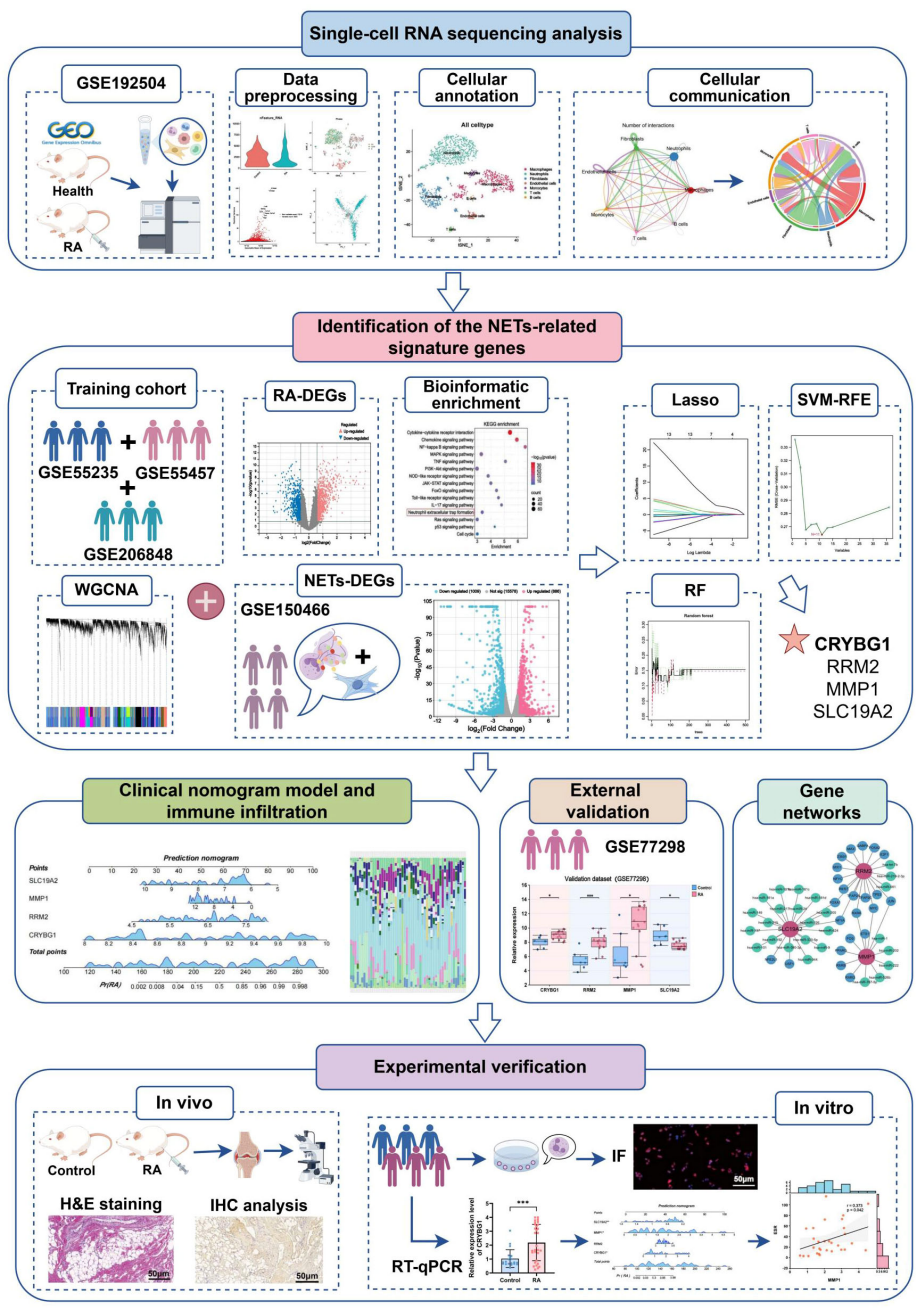
The flowchart depicting the investigation procedure of this study.

Recently, systems bioinformatics, combining biology, computer science, and statistics, has been widely applied to explore the mechanisms underlying various disease phenomena (21). Single-cell RNA sequencing (scRNA-seq) is a revolutionary technology for high-dimensional mapping of diseased organisms at single-cell resolution, revealing cellular heterogeneity, developmental processes, and interactions in specific tissues (22, 23). Machine learning algorithms can accurately identify specific expression patterns in gene expression profiles, prioritize key regulatory genes, and construct predictive models for identifying disease risk and treatment response (24). Based on these, we first analyzed synovial tissue single-cell mapping in RA, revealing neutrophil-fibroblast communication networks. Subsequently, we integrated multiple datasets and multiple machine algorithms to identify and develop a valuable RA diagnostic model based on hub genes associated with NETs. The specific investigation procedure are displayed in detail in **Figure 1**. Notably, the expression of these pivotal genes was not only validated in single-cell mapping, but also fully explored *in ex vivo* and *in vivo* experiments and clinical data, which are expected to provide new insights into the immune mechanisms and precise diagnostic strategies for RA.

## 2 Materials and methods

### 2.1 Data sources and preparation

For this study, scRNA-seq, microarray, and bulk RNA-seq datasets were obtained from the Gene Expression Omnibus database (GEO, https://www.ncbi.nlm.nih.gov/geo/). The dataset GSE192504, involving sequencing data from the knee joints of collagen-induced arthritis (CIA) mice and healthy control mice, was used for single-cell level analysis. Three microarray datasets, GSE55235, GSE55457, and GSE206848, containing a large number of human synovial tissue samples, were selected as training cohorts for the machine learning program, providing a solid foundation for model development. In cases where multiple probes identified the same gene, we calculated the median as the final expression and normalized the expression matrix. We applied the “Combat” algorithm in SVA to normalize the expression values of different batches or platforms, ensuring robust data. We also used the Uniform Manifold Approximation and Projection (UMAP) algorithm to assess the success of batch effect removal. The dataset GSE150466, which includes extensive RNA sequencing data from FLS treated with NETs for 48 h, was used to screen for genes closely associated with NETs. Additionally, GSE77298 was selected as an external validation cohort to ensure that our results could be confirmed using different datasets. **Supplementary Table S1** lists the details of all datasets involved.

### 2.2 Single-cell data processing and cellular annotation

Single-cell RNA sequencing data analysis was primarily performed using the “Seurat (v 5.1.0)” package (25). Initially, cells with fewer than 200 genes and fewer than three cells covered by the genes were excluded. We performed standard data preprocessing procedures to ensure the high quality of the data, and the quality control criteria included (1) RNA features > 200 and < 5000, (2) unique molecular identifiers (UMIs) of not less than 600, (3) percentage of mitochondrial genes < 10%, and (4) percentage of hemocyte genes < 1%. Subsequently, the “SCTransform” function was used to normalize data, scale it, and remove cell cycle effects; 3000 highly variable genes were identified for further analysis. We also performed principal component analysis (PCA) to reduce dimensionality, and “Harmony” corrected batch effects in the single-cell analysis, selecting the top 30 principal components for integration. We used t-distributed stochastic neighborhood embedding (t-SNE) for dimensionality reduction and visualization of single-cell data. Next, the “FindAllMarkers” function was used to identify differentially expressed marker genes in each cluster, and we manually labeled cell types based on a combination of SingleR auto-annotation as previously reported in the literature. Further visualization of hub genes was achieved using the “FeaturePlot” function.

### 2.3 Intercellular communication analysis

we used the “CellChat (V1.6.1)” package to visualize the cellular communication network and reveal key signaling pathways, specific ligands, and receptors to explore the communication patterns between different cells in the RA microenvironment. Special attention was paid to interactions between neutrophils and other cells, particularly fibroblasts.

### 2.4 Identification of differently expressed genes

The R package “limma” was used to identify genes showing significant differential expression in different comparison sets as visualized by volcano plots. Analyses were conducted in the merged training cohort of GSE150466 and GSE77298 with screening conditions of *P* < 0.05 and |logFC|> 1.5. NETs Related genes were defined as overlapping genes from training set-based DEGs, key module genes, and GSE150466-based DEGs, as shown in the UpSet plot.

### 2.5 Bioinformatic enrichment analysis

In order to identify major biological terms and related molecular pathways, Gene Ontology (GO) and Kyoto Encyclopedia of Genes and Genomes (KEGG) enrichment analyses were primarily performed using the Metascape (https://metascape.org/) website with parameters set to “H species” and *P* < 0.05 as the threshold for significant enrichment. Biological processes (BP), cellular components (CC), and molecular functions (MF) constituted the GO annotation system. In addition, we used the R packages “ClusterProfiler” and “enrichplot” to perform gene set enrichment analysis (GSEA). It is commonly used to estimate differences in BPs and pathways between samples in expression datasets (26), and p < 0.05 and FDR < 0.25 are considered statistically significant enrichments.

### 2.6 Weighted gene correlation network analysis

In the training cohort, the R package WGCNA was utilized to construct gene co-expression networks, a systems biology approach that identifies gene co-expression modules linked to clinical phenotypes through hierarchical clustering (27). First, we deleted 50% of the median absolute deviation (MAD) genes while removing outlier samples from the cluster tree. Based on the scale-free topology criterion, the “pickSoftThreshold” function was used to select the appropriate soft-threshold power (β) to compute the neighbor relationship. Subsequently, we constructed an adjacency matrix and converted it into a topological overlapping matrix (TOM). Using the dissimilarity measure of TOM, we performed average chained hierarchical clustering, where each module was required to contain at least 30 genes; the sensitivity was set to 3, the module-merging threshold was 0.25, a clustering tree was constructed, and the genes were classified into different modules with random colors. Furthermore, we collected candidate genes by calculating the correlation coefficients between the modules and clinical traits to select the modules with the most significant module-trait relationships. Correlations between gene significance (GS) and module affiliation (MM) values for all genes within a module were also generated and evaluated, and significant genes were selected for subsequent analyses.

### 2.7 Chromosomal location of genes

The R package “RCircos” was used to map the locations of the featured genes in the chromosomes, with the gene location information referenced from previously organized literature.

### 2.8 Machine learning

Here, we applied the Least Absolute Shrinkage and Selection Operator (LASSO) regression, Support Vector Machine Recursive Feature Elimination (SVM-RFE), and Random Forest (RF) algorithms to identify the most important genes associated with NETs. LASSO, a machine learning technique combining variable selection and regularization, is implemented through the “glmnet” package and executed to identify the hub genes in the training set, using 10-fold cross-validation for selection and lambda with minimum binomial deviation as the optimal value (28). SVM-RFE selects the best subset of features by eliminating noisy or redundant features, reducing the dimensionality of the data, and then evaluating the accuracy, primarily performed by “e1071,” “kernlab” and “caret” packages (29). The RF algorithm, on the other hand, utilizes integrated learning to generate a decision tree to evaluate the importance of the feature genes (30). Overlapping genes identified by the three algorithms were defined as central genes for subsequent research and validation.

### 2.9 Development and validation of nomogram

Based on the four central genes, the R package “rms” was applied to construct a nomogram model for predicting the risk of RA, which mainly consists of “Points” indicating the corresponding values of the candidate genes and “Total Points” displaying the sum of the scores of all the genes. Furthermore, the “nomogramFormula” and “pROC” packages were used to plot the receiver operating characteristic (ROC) curves to evaluate the predictive performance of the nomogram and the pivotal genes in the diagnosis of RA. The “calibrate” function and the “rmda” package were used to construct calibration curves, decision curve analysis (DCA), and clinical impact curves (CIC) to validate the clinical validity of the nomogram.

### 2.10 Immune infiltration analysis

We applied the “CIBERSORT” algorithm to determine the distribution ratio of 22 immune cells in the synovial tissues of RA and control groups in the training set (31), characterizing the immune landscape of RA. The results were presented in stacked histograms and box plots using the “ggplot” package. Furthermore, we applied the Spearman method to analyze the correlation between NETs-related hub genes and immune cells, as well as the correlation between different immune cells, and visualized the results in a combined correlation heatmap using the “ggcor” package.

### 2.11 Targeted network construction

We utilized the NetworkAnalyst (http://www.networkanalyst.ca) platform for the complex targeting analysis of hub genes. The TF-Gene interaction network was based primarily based on the ENCODE database; the gene-miRNA interaction network relied on the miRTarBase database, and the TF-miRNA co-regulation network also utilized the miRTarBase database. miRNA co-regulatory networks were constructed using the RegNetwork database and visualized with Cytoscape software.

### 2.12 Establishment of animal model

Male Sprague-Dawley rats aged 6-8 weeks (180 ± 20 g) were purchased from Huachuang Cigna Pharmaceutical Technology Co., Ltd. (Jiangsu, China) and housed in the Experimental Animal Research Center of the First Affiliated Hospital of Anhui University of Chinese Medicine. All experimental procedures adhered strictly to the National Institutes of Health Guide for the Care and Use of Laboratory Animals and were approved by the Laboratory Animal Ethics Committee of the Anhui University of Traditional Chinese Medicine (AHUCM-rats-2023020). The rat model of adjuvant-induced arthritis (AA) was established according to a previously published protocol (32). In addition, both control and model mice were fed the same food and kept in the same feeding environment until their joints were collected for histological analysis.

### 2.13 Histological assessment

The knee joints of the rats were collected, fixed with 4% paraformaldehyde, decalcified, embedded, and sectioned. Hematoxylin and eosin (H&E) staining was subsequently performed and thoroughly examined under a light microscope.

### 2.14 Immunohistochemistry

The synovial tissue was sequentially embedded in paraffin, sectioned, dewaxed, and hydrated. Subsequently, the tissues were treated with the target repair solution EDTA buffer in an autoclave under high pressure for antigen repair, incubated with 3% H_2_O_2_ for 20 min for inactivation, and then washed with water. Next, primary antibodies CRYBG1 (1:300, bs-9093R, bioss), RRM2 (1:300, bs-7133R, bioss), MMP1 (1:100, ab52631, abcam), and SLC19A2 (1:300, bs-10738R, bioss) were added dropwise to the sections and placed in the incubator at 37°C for 60 min. The reaction mixture was then incubated with the secondary antibody for 30 min and stained with DAB. Finally, reaction images were obtained using microscopy and quantified with ImageJ software.

### 2.15 Clinical sample collection

Thirty patients with rheumatoid arthritis (RA) who were hospitalized in the Department of Rheumatology at the First Affiliated Hospital of Anhui University of Chinese Medicine between January 2022 and June 2024 were included in the study; 20 age- and sex-matched healthy volunteers were recruited from the physical examination center. Peripheral blood samples and clinical data were obtained from the patients for further experimentation and analysis. The clinical data were mainly included neutrophil to lymphocyte ratio, erythrocyte sedimentation rate (ESR), hypersensitive C-reactive protein (hs-CRP), Interleukin 6 (IL-6), antistreptolysin O (ASO), rheumatoid factor (RF), anti-cyclic citrullinated peptide antibody (anti-CCP), immunoglobulin (Ig) A, IgG, IgM, complement component 3 (C3) and complement component 4 (C4). This study was approved by the Ethics Committee of the hospital, and informed consent was obtained from all subjects (Ethics Number: 2023AH-52).

### 2.16 Neutrophil isolation and immunofluorescence staining

Human peripheral neutrophils (PMNs) were isolated using Percoll’s continuous density gradient separation method, and the lower white membrane layer (i.e., the peripheral neutrophil layer) was aspirated, washed with 10 ml of PBS, and centrifuged for last use. Phorbol 12-myristate 13-acetate (PMA) was used to stimulate the formation of NETs for 4 hours at a concentration of 5 µmol/µL. Next, immunofluorescence staining was performed to examine NET formation. The PMNs were fixed in 4% paraformaldehyde and incubated with 0.5% TritonX-100 for 30 min for permeabilization. Then, they were incubated with anti-MPO antibody (1:200, bs-4943R, Bioss) and the anti-NE antibody (1:400, ab310335, abcam) for 60 min in a 37°C incubator. The sections were then incubated with goat anti-rabbit IgG-labeled secondary antibody (1:400) for 30 min and washed three times with PBS before blocking with an anti-fluorescence quenching blocker containing 4′,6-diamidino-2-phenylindole (DAPI). Finally, the NETs were imaged using fluorescence microscopy to assess their locations and numbers.

### 2.17 Real-time quantitative polymerase chain reaction

Total RNA was extracted from cell lysates using TRIzol reagent according to the manufacturer’s instructions and reverse transcribed into cDNA using a reverse transcription kit. Taq SYBR Green qPCR Premix (Universal) was used to perform a fluorescence quantitative PCR reaction. We chose to use β-actin as a normalized internal control and applied the 2-ΔΔCt method for calculating the relative mRNA level. All the primers used in this study are listed in **Supplementary Table S2**.

### 2.18 Statistical analysis

All statistical analyses were performed using R software (version 4.4.1), GraphPad Prism 9, and SPSS 26.0. Two independent sample t-test and paired Student’s t-test were used to compare the two groups of samples. Univariate logistic regression model analysis was used to identify pivotal genes of diagnostic value. Correlations between variables were assessed using Pearson’s or Spearman’s method. All data are expressed as mean ± standard deviation, and statistical tests were two-sided. Statistical results are reported as p-value with significance at *P* < 0.05.

## 3 Results

### 3.1 Single-cell landscape of the RA synovial microenvironment

**Figure 2:**
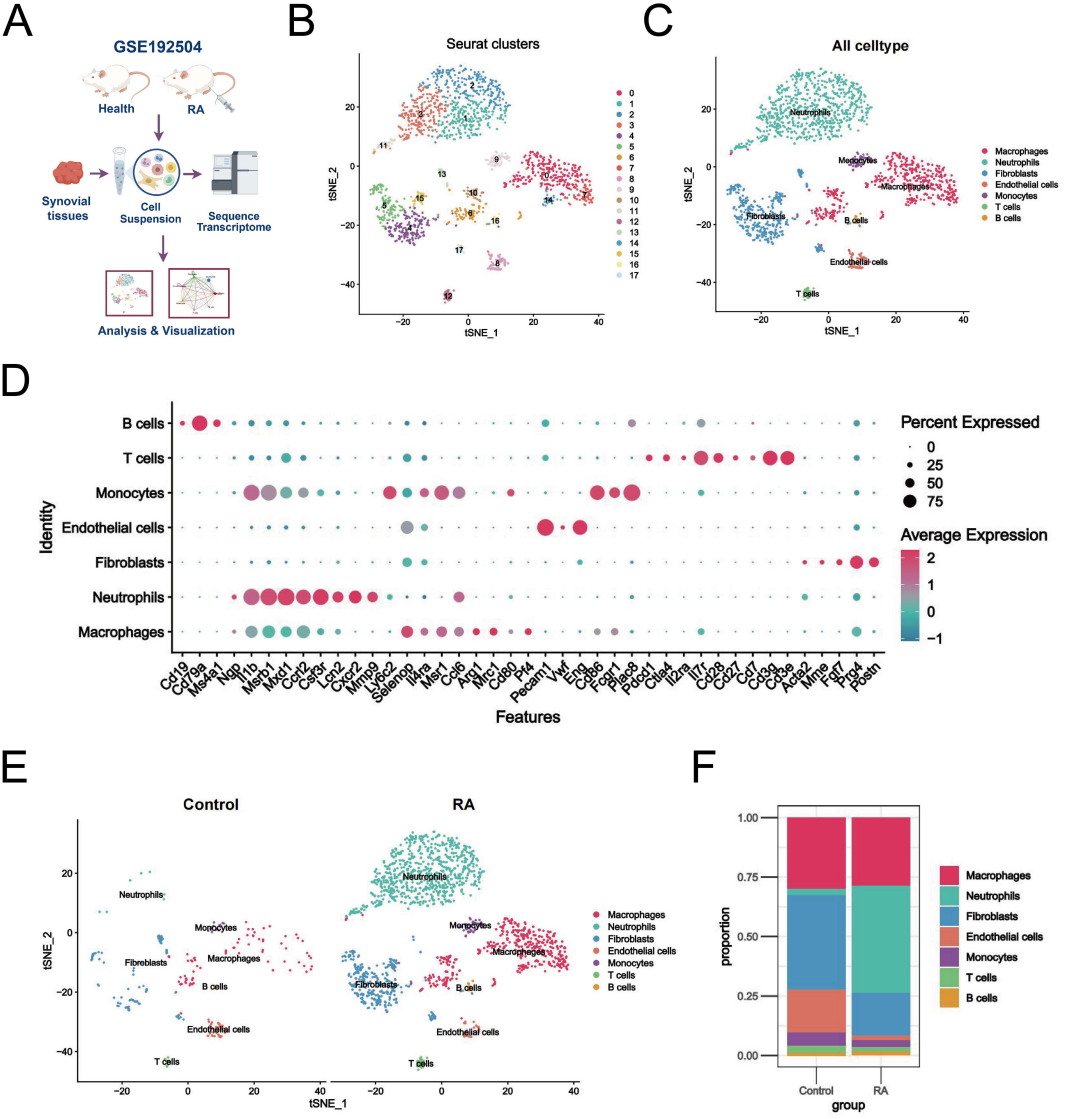
Single-cell landscape of the RA synovial microenvironment. **(A)** Flowchart of single-cell analysis. **(B)** t-SNE plot showing 18 clusters (0-17) identified from the GSE192504 dataset by dimensionality reduction clustering analysis. **(C)** Clusters were annotated into 7 cell types based on SingleR and literature. **(D)** Dot plots of marker gene expression levels annotated by different cell types. **(E)** Differences in cell distribution and expression between RA and healthy controls. **(F)** Stacked plot of the proportion of abundance of each cell type in synovial tissues of RA and healthy controls.

Initially, two synovial tissue samples were selected for single-cell analysis using the GSE192504 dataset (**Figure 2A**). We checked the mitochondrial gene and blood cell ratios for quality control (**Supplementary Figures S1A, B**) to ensure the completeness and reliability of the single-cell transcriptome dataset analysis. Ultimately, 260 cells derived from normal tissues and 1,800 eligible cells derived from CIA tissues were included in the downstream analysis. Given the potential heterogeneity of cell cycle phases in the dataset, we assessed the consistent distribution of these phases within the samples using a cell cycle scoring technique (**Supplementary Figure S1C**). Filtered data were analyzed using the HARMONY method, focusing on highly variable genes (**Supplementary Figures S1D-H**). Next, we categorized all cells into 18 clusters using t-SNE (**Figure 2B**). Based on SingleR and the related literature, we annotated the cells into seven different types: macrophages, neutrophils, fibroblasts, endothelial cells, monocytes, T cells, and B cells (**Figure 2C**). Dot plots show the expression levels of the marker genes associated with each cell population (**Figure 2D**). Relative to healthy controls, there was a significant increase in macrophages, neutrophils, fibroblasts, and monocytes and a relative decrease in endothelial cells in the RA synovium (**Figure 2E**). Neutrophils exhibited the most significant increase in abundance (**Figure 2F**). These findings suggest a heterogeneous landscape of the synovial microenvironment between normal and RA samples.

### 3.2 Kinetic analysis of intercellular communication in the RA synovial microenvironment

**Figure 3:**
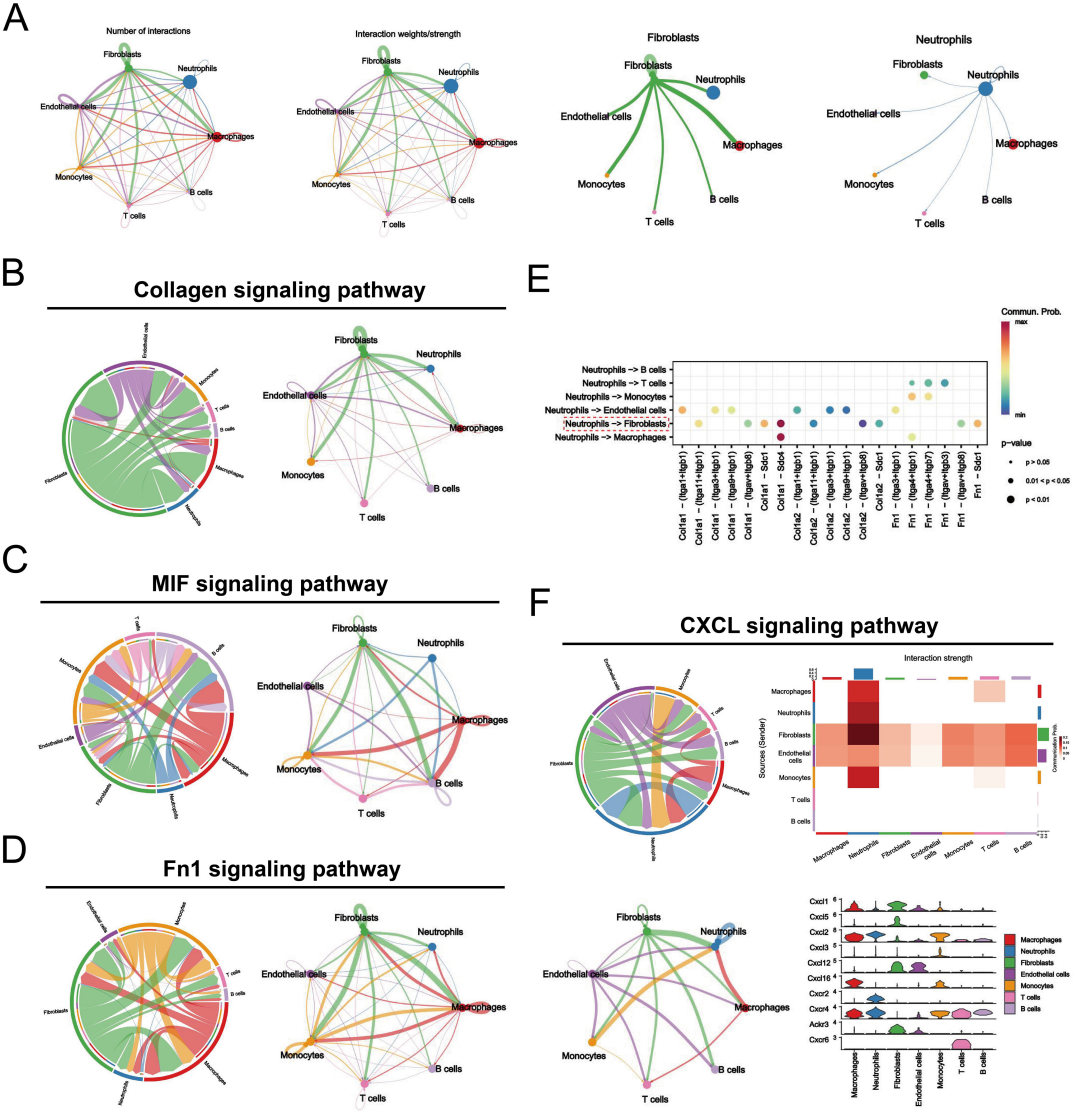
Kinetic analysis of intercellular communication in the RA synovial microenvironment. **(A)** Circle plots plotting the weights of interactions between the seven major cellular subclusters in the RA group, with fibroblasts and neutrophils shown in detail. **(B-D)** Graphical representation of the three major signaling pathways (Collagen signaling pathway, MIF signaling pathway, and Fn1 signaling pathway) by which neutrophils and fibroblasts interact, including chordal diagrams (left) and circle diagrams (right) demonstrating the process of cellular interactions. **(E)** Dot plots highlighting ligand-receptor pairs between neutrophils and other cells in the RA microenvironment, with the size of the dots representing the p-value of pathway involvement and colored by the probability of communication. **(F)** A graphical representation of the CXCL signaling pathway, consisting of chord and circle diagrams demonstrating the process of cellular interactions, an exploratory heatmap highlighting the strength of interactions, and a violin plot exploring the level of gene expression.

Dissecting the interactions between neutrophils and other cell populations in the synovial inflammatory microenvironment, especially communication with fibroblasts, will help to further elucidate the mechanisms of NETs formation in RA. The evaluation of the “CellChat” package reveals that both neutrophils and fibroblasts sent a large number of signals to other cells, suggesting that their high activation is associated with the development of RA (**Figure 3A**). Furthermore, we conducted an in-depth exploration of key ligand pathways in the cellular communication network. The results showed that the major pathways extending from neutrophils to fibroblasts were the collagen, MIF, and Fn1 signaling pathways (**Figures 3B–D**). We also found that these interactions mainly occurred through Col1a1-Sdc4 interactions (**Figure 3E**). In addition, the CXCL signaling pathway, which regulates the immune response and inflammation, is known to play an important role in the RA microenvironment. We found that neutrophils, which act as signal initiators and influencers, dominate this pathway (**Figure 3F**). Intriguingly, CXCL2-CXCR2 contributed most to this pathway, suggesting that it may be critical for promoting crosstalk between neutrophils and other cells in the RA microenvironment, which warrants further investigation. Overall, these findings emphasize that neutrophils affect fibroblasts in the RA microenvironment through multiple signaling pathways and associated ligands.

### 3.3 Differential gene identification and biological characterization of RA

**Figure 4:**
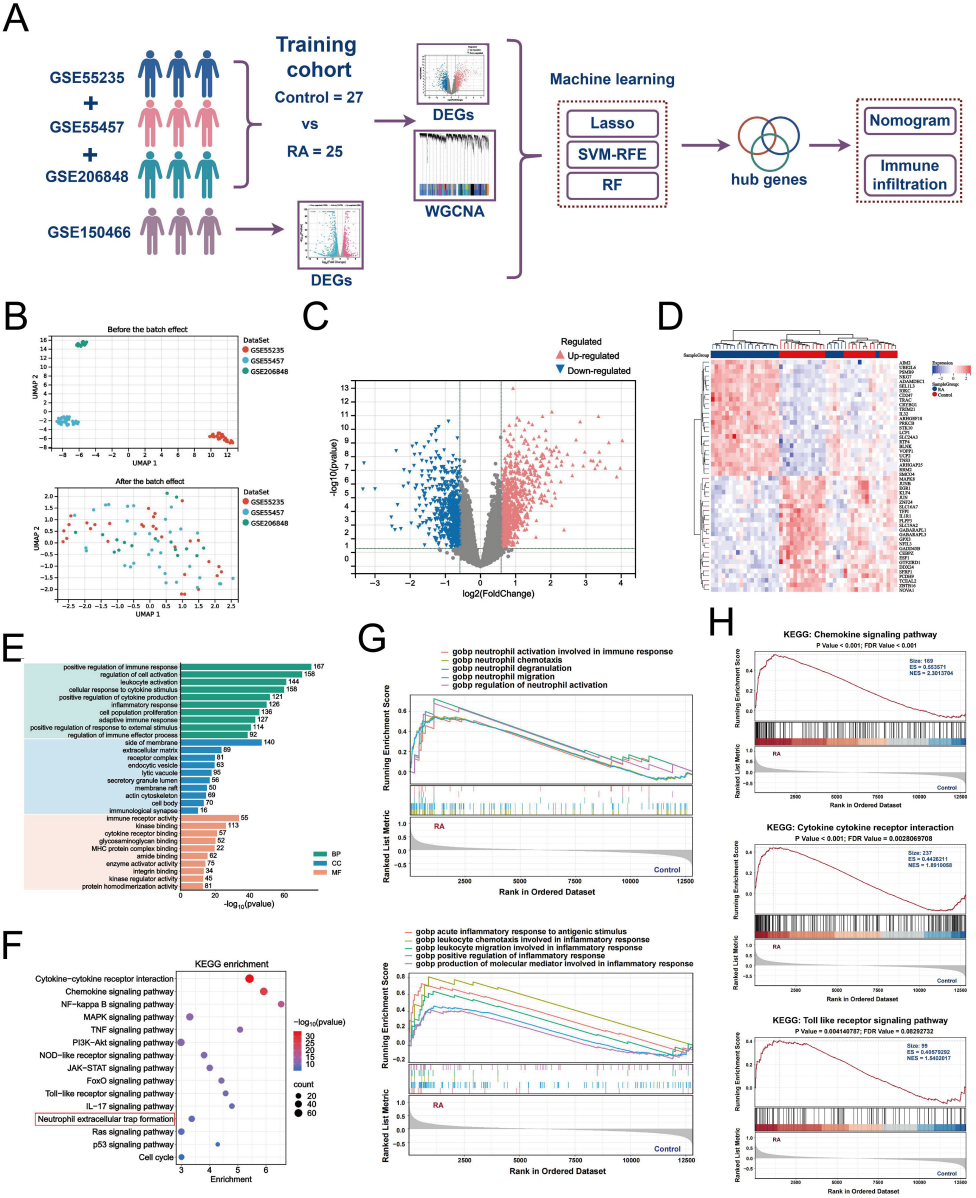
Differential gene identification and biological characterization of RA. **(A)** Schematic of batch RNA sequencing analysis. **(B)** UMAP-based analysis showing the distribution of sample expression before and after de-batch effects for GSE55235, GSE55457, and GSE206848. **(C)** Volcano plot showing the differentially expressed genes (|logFC| > 1.5 and *P* < 0.05) between RA and control in the training cohort merged by the three data sets. **(D)** Heatmap showing the top 50 up-/down-regulated genes in the differential expression analysis between the two samples. **(E)** Bar graph demonstrating the results of GO function analysis of RA key genes. **(F)** Bubble graph showing the KEGG analysis results of RA key genes. GSEA results of GO enrichment **(G)** and KEGG enrichment **(H)** based on gene expression levels.

Considering that NETs effectively contribute to the synovial pathological microenvironment of RA, we next identified key genes and functional features associated with NETs (**Figure 4A**). We merged and batch-normalized the three datasets — GSE55235, GSE55457, and GSE206848 — downloaded from GEO to broadly and comprehensively explore key genes in a larger training cohort for subsequent analyses containing 25 synovial biopsies from RA patients and 27 synovial biopsies from matched healthy individuals (**Figure 4B**). Subsequently, 1419 DEGs were identified from the training cohort, of which 763 were upregulated and 656 were downregulated, based on the screening criteria of *P* < 0.05 and |logFC| > 1.5 (**Figure 4C**). The top 50 genes were selected from the up-down-regulated genes to create a heatmap (**Figure 4D**). Next, we performed GO and KEGG enrichment analyses of the DEGs to characterize RA function. GO analysis showed that these differential genes were mainly involved in positive regulation of the immune response, regulation of cell activation, leukocyte activation, cellular response to cytokine stimulus, and positive regulation of cytokine production, among other immune-related biological processes (**Figure 4E**). KEGG enrichment analysis identified the top 10 significantly enriched pathways as follows: cytokine-cytokine receptor interaction, chemokine signaling pathway, NF-kappa B signaling pathway, MAPK signaling pathway, TNF signaling pathway, neutrophil extracellular trap formation, and other inflammation-related pathways (**Figure 4F**). In addition, we performed GSEA enrichment analysis to explore the differences in gene function between the RA synovium and normal samples. In GO-enriched GSEA, neutrophil migration, chemotaxis, and degranulation, as well as the acute inflammatory response to antigenic stimuli and positive regulation of the inflammatory response, were significantly activated in RA (**Figure 4G**). Notably, in KEGG-enriched GSEA, the chemokine signaling pathway, cytokine receptor interaction, and toll-like receptor signaling pathway, which are pathways were significantly enriched in RA (**Figure 4H**). These results strongly suggest that neutrophil migration and inflammatory responses are involved in the progression of RA synovial pathology.

### 3.4 Construction of WGCNA and identification of key module genes in RA

**Figure 5:**
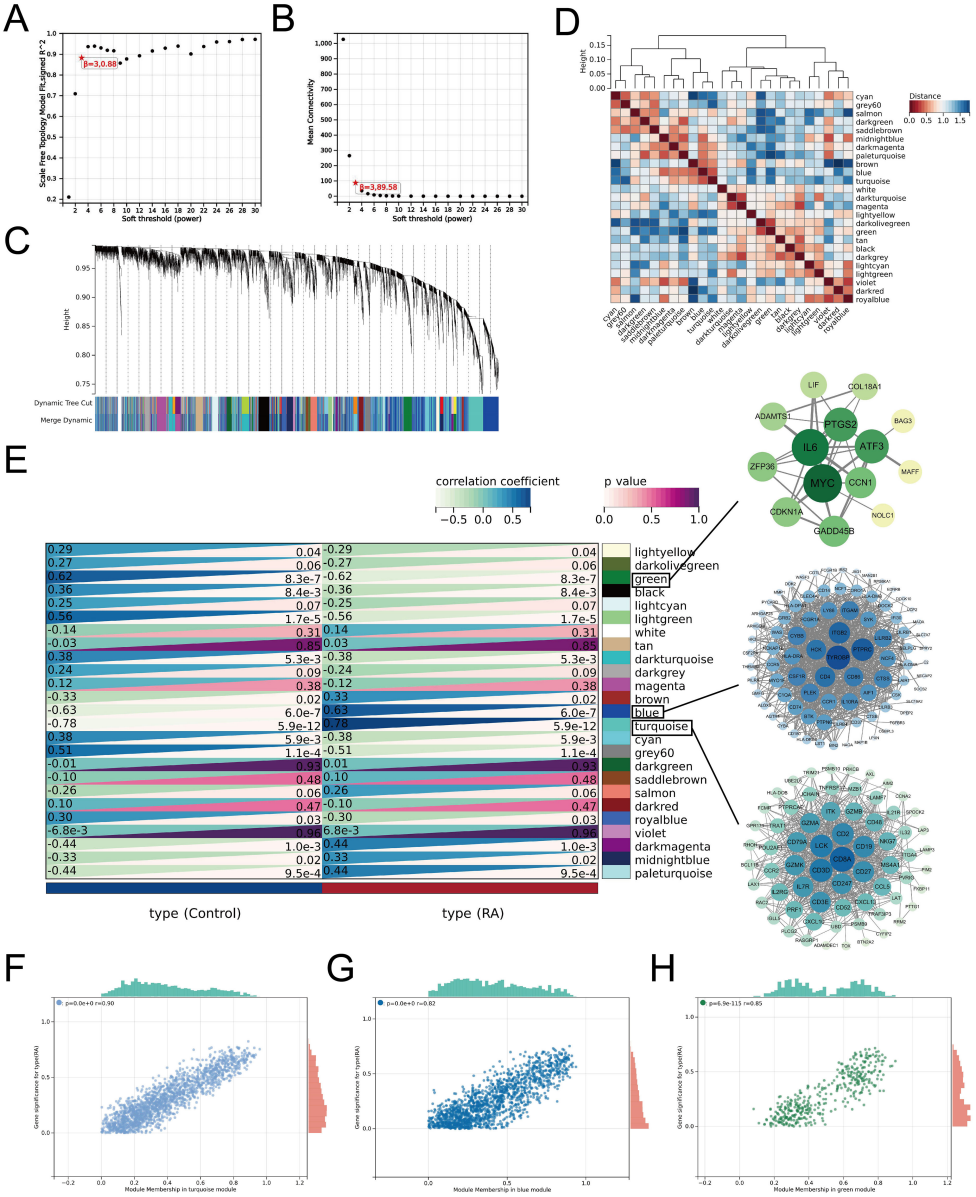
Construction of WGCNA and identification of key modular genes in RA. **(A)** Estimation of scale-independent indices for different soft-threshold powers. **(B)** Estimation of average connectivity for different soft threshold powers. **(C)** Gene hierarchy clustering dendrogram in which different modules are indicated by different colors. **(D)** Heatmap for inter-module correlation analysis. **(E)** Relationships between module-characterized genes and clinical traits in the RA group and normal control group, with numbers in the modules representing correlation coefficients and p-values. Further screening of candidate hub genes within modules (with GS > 0.1 and MM > 0.8 as thresholds) was visualized by Cytoscape. Scatter plots were generated to show the association between GS and MM within the turquoise module **(F)**, blue module **(G)**, and green module **(H)**, respectively.

We constructed gene co-expression networks using WGCNA to mine the key gene modules associated with RA accurately. Based on module independence and average connectivity, we set the soft threshold parameter β to 3 to ensure the construction of a scale-free gene network, in which the corresponding R^2^ was 0.88, and the average connectivity was very high (**Figures 5A, B**). Next, we constructed a gene hierarchy clustering dendrogram and identified 25 gene co-expression modules (**Figure 5C**). The correlations among the modules are shown in **Figure 5D**. Furthermore, we identified associations between the genes in each module and the clinical traits using correlation analysis. In the heat map of module-clinical trait relationships, the turquoise (r = 0.78), blue (r = 0.63), and green modules (r =-0.62) showed the strongest correlation with RA (**Figure 5E**). The scatter plots showed strong correlations between GS and MM in all three modules (**Figures 5F–H**). Subsequently, we screened a total of 204 center module genes for subsequent analyses with GS > 0.1 and MM > 0.8.

### 3.5 Identification of NETs-related hub genes in RA

**Figure 6:**
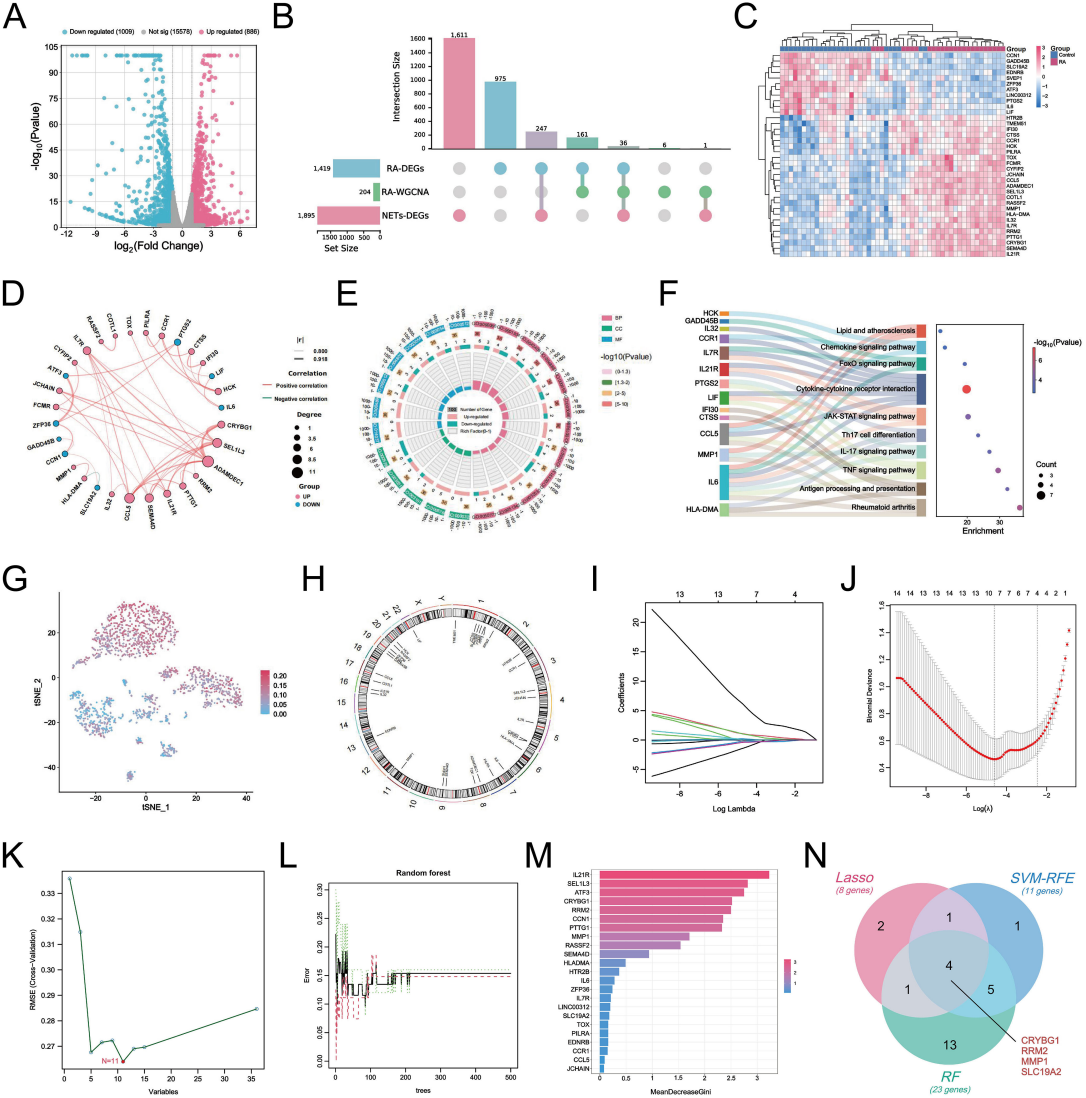
Identification of NETs-related core hub genes in RA. **(A)** Volcano plots demonstrating differential genes between NETs-stimulated and unstimulated FLS in the GSE150466 dataset (|logFC| > 1.5 and *P* < 0.05). **(B)** UpSet crossover plot showing the distribution of overlapping genes between RA-DEGs, co-expressed key module genes, and NETs-DEGs. **(C)** Heatmap showing the expression differences of the 36 crossovers characterized genes in the tested cohort. **(D)** Circle correlation connectivity plot of co-expression among the 36 characterized genes. **(E)** Circle plot showing the results of GO enrichment analysis of 36 feature genes, including 12 BP terms, 5 CC terms and 7 MF terms. **(F)** Sankey bubble plot showing the top 10 most significant KEGG enrichment results. **(G)** t-SNE plot showing the distribution of 36 characterized genes in different cells at the single-cell level. **(H)** Circle plot of 36 characterized genes localized on chromosomes. **(I)** Eight candidate hub genes were obtained based on LASSO regression and 10-fold cross-validation. **(J)** Generation of coefficient profiles for log(lambda) sequences in the LASSO model, the vertical dashed line is the optimal log(λ) value. **(K)** Establishment of 11 potential hub genes after identification by SVM-RFE algorithm. **(L)** Random forest tree plot depicting the error rate versus the number of classification trees, where red, green, and black points represent RA samples, normal group samples, and all samples, respectively. **(M)** Bar chart showing the top 23 genes ranked based on importance scores. **(N)** Venn diagram showing the identification of four final NETs-related core genes by the three algorithms described above.

we performed differential gene identification on the GSE150466 dataset, obtaining a total of 1,895 NET-associated DEGs (**Figure 6A**), comprising 886 upregulated (blue) and 1,009 downregulated (red) genes to explore the key mechanisms involved in NET formation. Subsequently, we intersected the above DEGs with RA-associated DEGs and key module genes and obtained 36 characteristic hub genes (**Figure 6B**). The heatmap shows the differences in the expression of these hub genes in the training set (**Figure 6C**). The correlation loop plot demonstrated that all differentially expressed genes exhibited strong regulatory associations with each other (**Figure 6D**). Comprehensive gene annotation and functional enrichment analyses were performed to elucidate the potential functions of these 36 key genes. Based on GO enrichment analysis, 12 BP terms, 5 CC terms, and 7 MF terms showed statistically significant enrichment within the core genes (**Figure 6E**). Notable BP enrichment included positive regulation of protein phosphorylation, regulation of the MAPK cascade, chemotaxis, positive regulation of cell activation, regulation of the inflammatory response, and negative regulation of the immune response. KEGG enrichment analysis showed that NET-related genes in RA were primarily involved in the TNF signaling pathway, IL-17 signaling pathway, Th17 cell differentiation, cytokine-cytokine receptor interaction, FoxO signaling pathway, and chemokine signaling pathway. Chemokine signaling pathways and other key pathways (**Figure 6F**). Here, we also revealed the distribution of these 36 characteristic genes in the RA synovial microenvironment by the single-cell mapping drawn previously (**Figure 6G**). We observed that these genes were significantly enriched in neutrophils, macrophages, and fibroblasts, with the most abundant expression found in neutrophils. **Figure 6H** shows the chromosomal locations of these genes. Furthermore, we integrated and applied three machine-learning algorithms to simplify the most critical feature variables. Based on the results of 10-fold cross-validation, LASSO identified eight potential candidate genes (**Figures 6I, J**). The SVM-RFE algorithm identified the 11 most important NET-related DEGs (**Figure 6K**). The RF algorithm determined the importance of the top 23 genes based on the error rate and number of classification trees (**Figures 6L, M**). By intersecting the results obtained from the three algorithms, a final list of four overlapping genes (**Figure 6N**) was obtained, namely CRYBG1, RRM2, MMP1, and SLC19A2, which will be investigated as pivotal genes in our future studies.

### 3.6 Development of NETs-associated nomogram and characterization of immune infiltration

**Figure 7:**
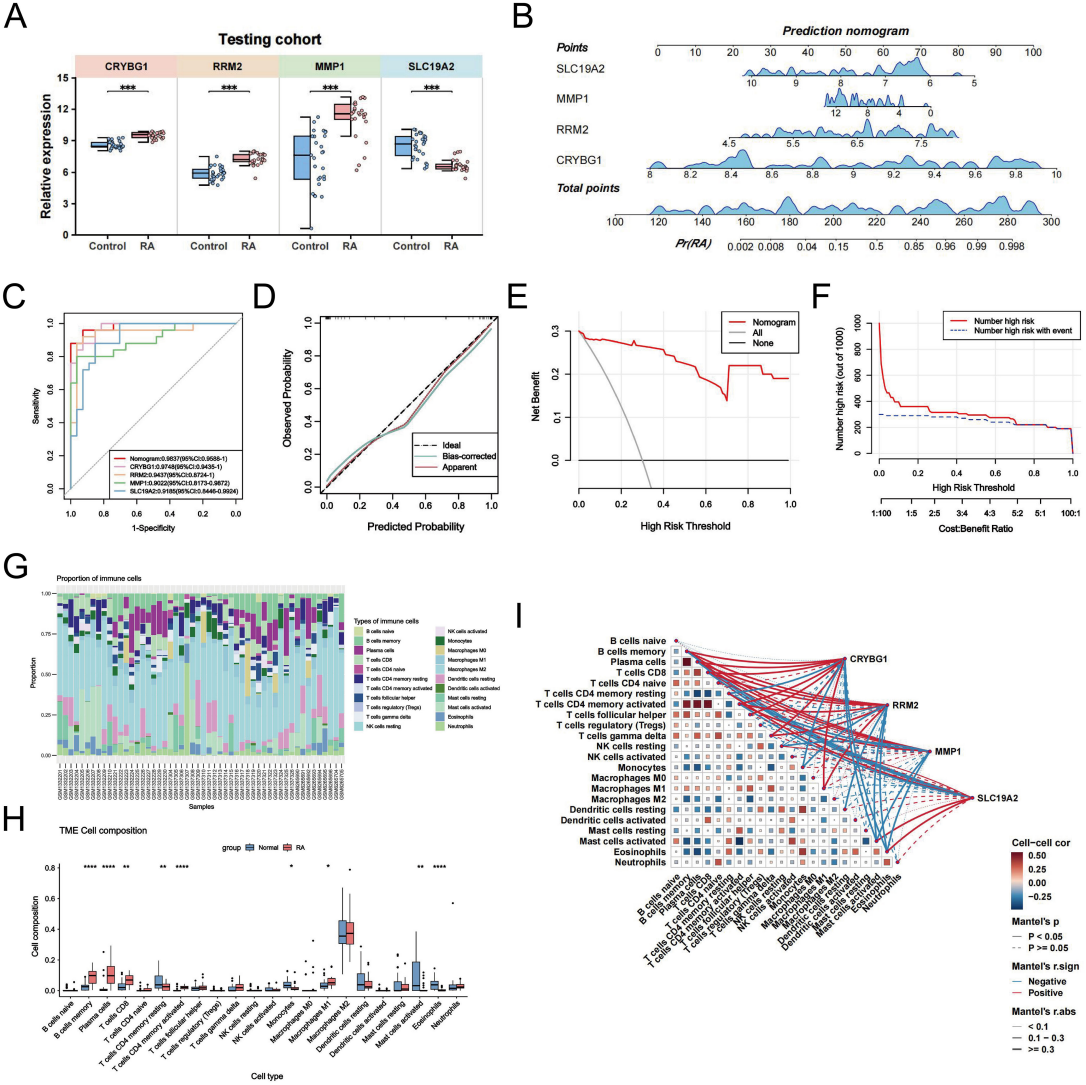
Construction of NETs-associated nomogram with immune infiltration characteristics. **(A)** Comparison of the expression levels of the four NETs-related hub genes between the control and RA groups in the training cohort. **(B)** Disease prediction score modeling based on four NETs-related hub genes for diagnosis of RA. **(C)** ROC curves of the nomogram model and the diagnostic performance of the four NETs-related hub genes within the model. **(D)** Calibration curves predicted by the nomogram model. **(E)** DCA curves are predicted by the nomogram model. **(F)** Clinical impact curves of the nomogram model. **(G)** The proportion of various immune cell infiltrates in the training cohort analyzed using the CIBERSORT algorithm. **(H)** Comparison of the levels of various immune cell infiltrations between the RA group and the control group in the training cohort. **(I)** Correlation heatmaps demonstrating the correlation between NETs-related hub genes and various infiltrating immune cells and between immune cells. Data are expressed as mean ± standard deviation. *P < 0.05, **P < 0.01, ***P < 0.001, ****P < 0.0001.

In the training cohort, CRYBG1, RRM2, and MMP1 were highly expressed in RA patients, whereas SLC19A2 was highly expressed in the normal group (**Figure 7A**). We developed an RA disease prediction model based on the four core genes in the training cohort to determine the diagnostic and predictive ability of the identified core genes. In the nomogram, each feature variable corresponded to a specific score, and the sum of all feature scores within each sample represented the probability of RA (**Figure 7B**). ROC curve analysis indicated that the AUC values of the candidate genes within the nomogram model were all > 0.9, suggesting that it was effective in distinguishing between RA patients and healthy individuals and that the nomogram had a superior predictive value when compared to single genes (**Figure 7C**). The calibration curves demonstrated that the predictive probabilities of the constructed nomogram diagnostic model were very close to the ideal curves, indicating that the model was predicted with good accuracy (**Figure 7D**). The DCA further illustrated that decisions based on the constructed nomogram model yielded a higher net benefit for the diagnosis of RA compared to interventions for all or no interventions (**Figure 7E**). Similarly, the CIC revealed that the estimated number of individuals identified as high-risk by the model converged with the number of true-positive events (**Figure 7F**). We also explored the patterns of immune cell infiltration in the RA training cohort. The results showed that the RA group exhibited a significantly increased abundance of memory B cells, plasma cells, T cells CD8, T cells CD4 + memory cells, and Macrophages M1 compared to the normal group, while T cells CD4 memory resting, monocytes, activated mast cells, and eosinophils showed a significant decrease in infiltration (**Figures 7G, H**). Correlation analysis revealed that the four identified central genes were strongly correlated with most immune cells (**Figure 7I**). Among these, CRYBG1 was strongly positively correlated with memory B cells and plasma cells, MMP1 and RRM2 were strongly negatively correlated with eosinophils, while SLC19A2 was strongly positively correlated with eosinophils. These results confirmed the previously identified inseparable relationship between the expression of hub genes and the pattern of immune infiltration in RA.

### 3.7 External validation of NETs-related hub genes and construction of target gene networks

**Figure 8:**
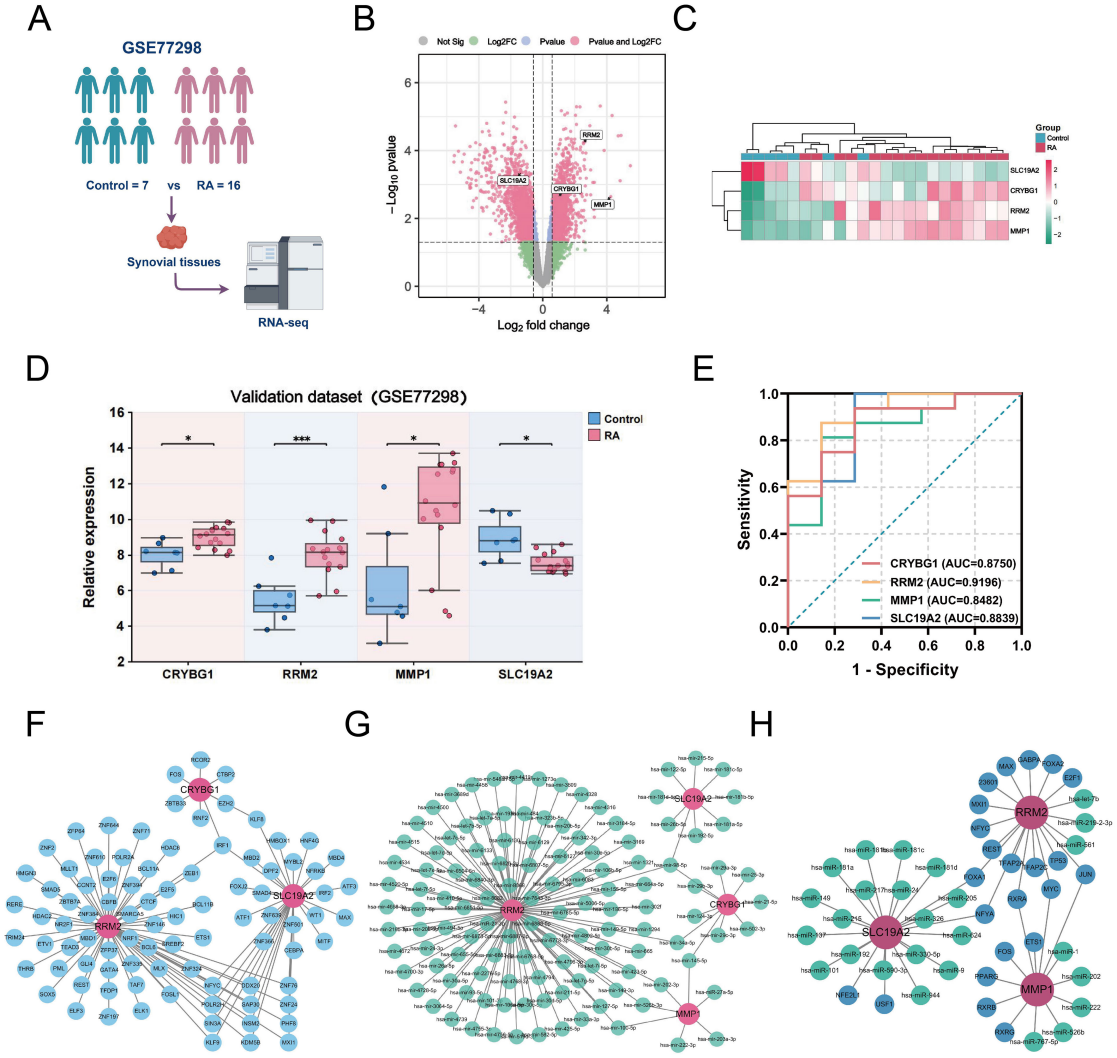
External validation of NETs-related hub genes and construction of target gene networks. **(A)** Schematic diagram of the analysis of the external validation dataset GSE77298. **(B)** Volcano plot showing the differentially expressed genes (|logFC| > 1.5 and *P* < 0.05) in the externally validated dataset GSE77298, in which 4 hub genes were labeled. **(C)** Heatmap showing the expression differences of the four hub genes between the two groups in the GSE77298 dataset. **(D)** Comparison of the expression levels of the 4 NETs-associated hub genes between the control and RA groups in GSE77298. **(E)** ROC curves highlighting the diagnostic performance of the 4 NETs-related hub genes in GSE77298. **(F)** Construction of TF-Gene interaction network. **(G)** Construction of Gene-miRNA regulatory network. **(H)** Construction of TF-Gene-miRNA co-regulatory network. Data are expressed as mean ± standard deviation. *P < 0.05, ***P < 0.001.

Considering the possible bias introduced by the merged dataset, we selected the external dataset GSE77298 to validate the expression of the four hub genes (**Figure 8A**). In a comparison of 16 RA and seven control samples, we identified 1414 upregulated DEGs and 2100 downregulated DEGs (**Figure 8B**). The heatmap further demonstrates a difference in hub gene expression between the two groups (**Figure 8C**). Specifically, CRYBG1, RRM2, and MMP1 showed higher levels in the RA group than in the normal group, whereas SLC19A2 showed significantly lower expression (**Figure 8D**), consistent with observation in the training cohort. The diagnostic value of the four hub genes in the external dataset was assessed using ROC curve analysis, which showed that the AUC values of all four genes exceeded 0.80, indicating good performance in identifying RA (**Figure 8E**). In addition, to confirm the hub genes and their associated upstream and downstream interplay factors, we constructed the TF-gene interaction network, gene-miRNA interaction network, and TF-miRNA co-regulation network (**Figures 8F–H**). We found that RRM2 occupied a central position in hub-gene interactions, IRF1 was a critical regulator, and has-mir-98-5p played an important co-regulatory role in the hub gene network.

### 3.8 Validation of hub gene expression in mouse models

**Figure 9:**
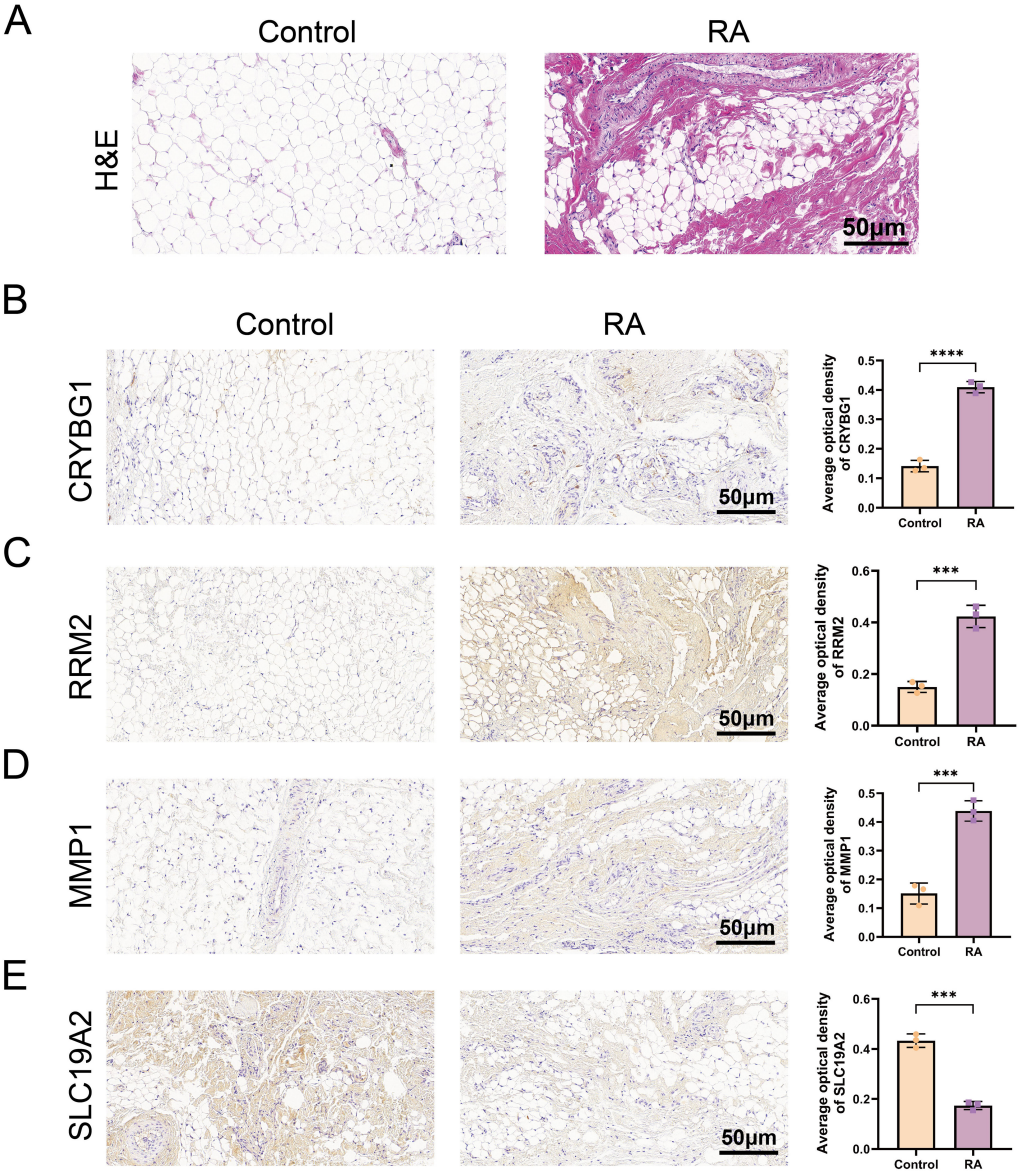
Validation of pivotal gene expression in the mouse model. **(A)** H&E-stained images of synovial tissues of a mouse model. Expression of CRYBG1 **(B)**, RRM2 **(C)**, MMP1 **(D)** and SLC19A2 **(E)** proteins in synovial tissues of mouse knee joints was detected by IHC (standard bar = 50 μm). Data are expressed as mean ± standard deviation. ***P < 0.001, ****P < 0.0001.

The results of the integrated bioinformatics analysis were validated by establishing an AA rat model. H&E staining showed that the synovial tissue in the model group exhibited thickening, disorganization, and significant inflammatory cell infiltration compared to the control group, confirming successful modeling for subsequent analysis (**Figure 9A**). We examined the expression of these four pivotal genes in the mouse synovium using IHC analysis. The IHC results showed a small amount of CRYBG1, RRM2, and MMP1 protein staining in the synovial tissues of the control group (brown color) and a significantly greater distribution and expression in the synovium of the model rats than in the controls (*P* < 0.0001). In contrast, SLC19A2 expression trended in the opposite direction (**Figures 9B–E**). These results are consistent with those obtained from the database.

### 3.9 Validation of NET formation and hub gene expression in clinical cohorts

**Figure 10:**
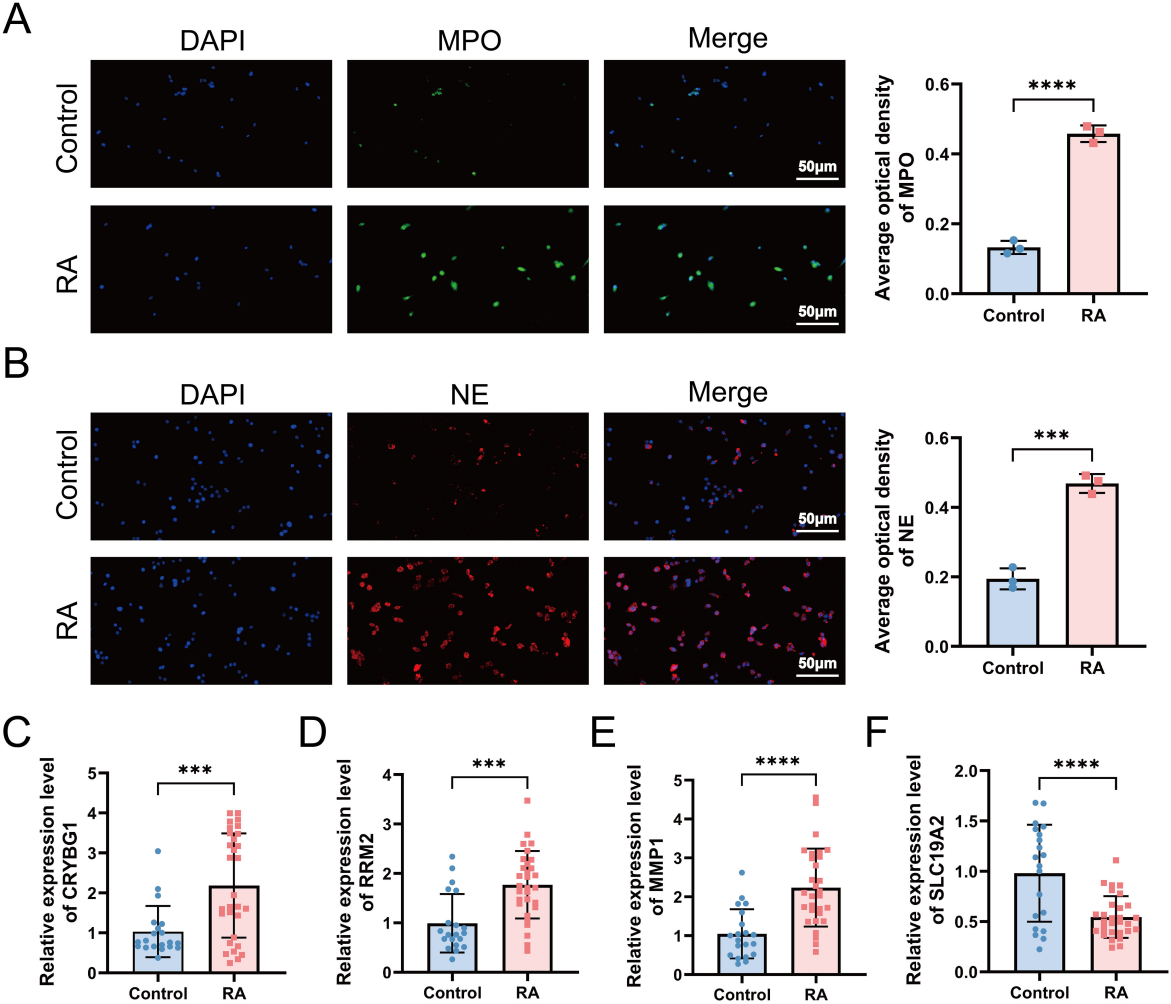
Validation of NETs formation and hub gene expression in a clinical cohort. **(A, B)** Immunofluorescence microscopy examination revealed the presence of NETs in RA, defined as MPO (green) and NE (red) (standard bar = 50 μm). RT-qPCR was applied to detect the expression levels of hub genes CRYBG1 **(C)**, RRM2 **(D)**, MMP1 **(E)** and SLC19A2 **(F)** in neutrophils. ***P < 0.001, ****P < 0.0001.

**Figure 11:**
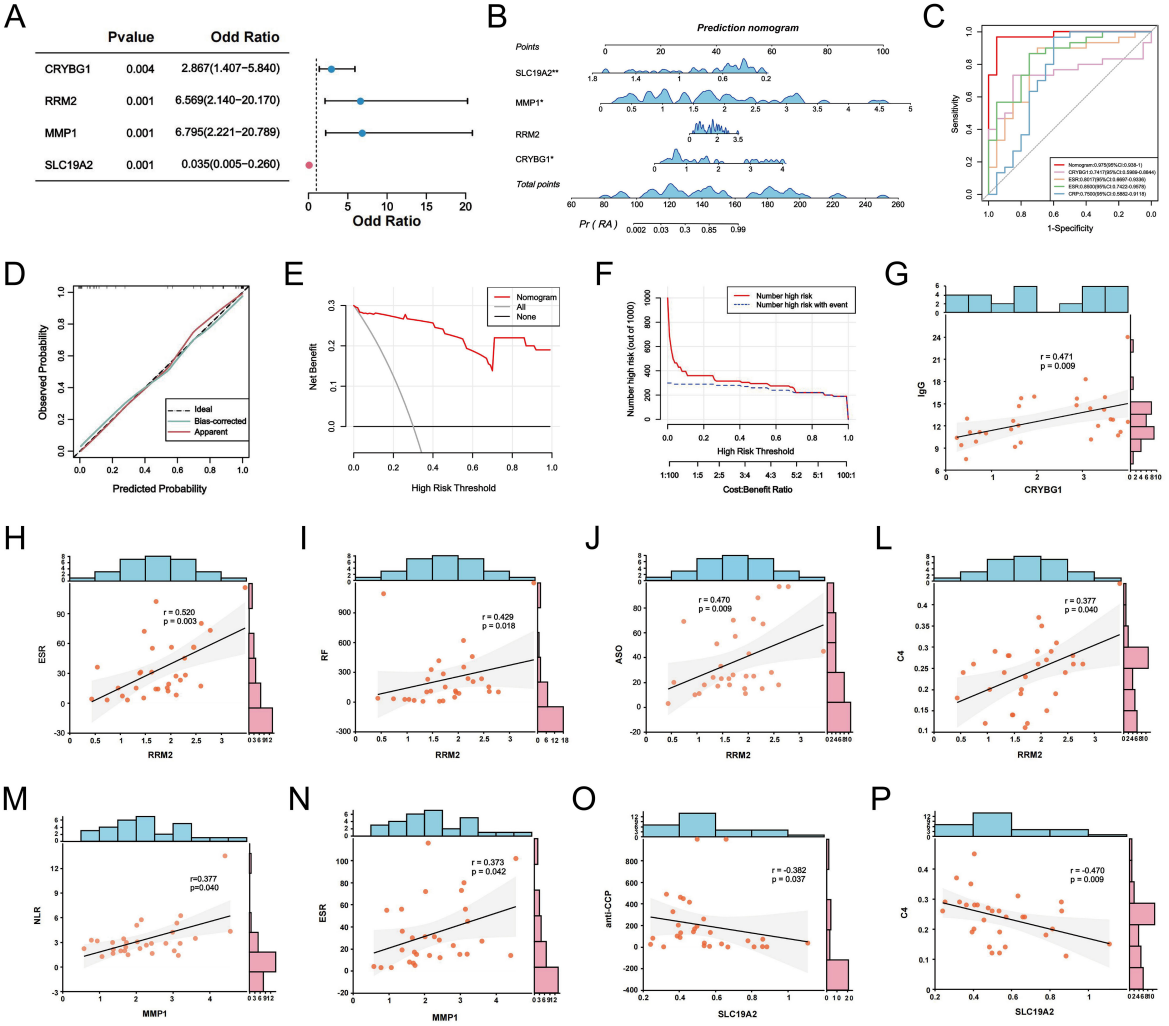
NETs-related hub genes are clinically relevant. **(A)** Forest plot of logistic regression analysis of hub genes in RA prediction. **(B)** The clinical RA prediction nomogram model is based on 4 NETs-related hub genes. **(C)** ROC curves of the nomogram model and the characteristic variables within the model. Calibration curves **(D)**, DCA **(E)** and CIC **(F)** were predicted by the nomogram model. **(G-P)** Correlation analysis between CRYBG1, RRM2, MMP1, and SLC19A2 and laboratory markers in RA patients. *P < 0.05, **P < 0.01.

We screened 30 patients with RA and 20 eligible healthy controls, as shown in **Supplementary Table S3**, to elucidate the relationship between NET-associated hub genes and clinical features of RA. IF images showed that the key markers for NETs in RA, MPO, and NE were primarily localized in neutrophil nuclei, with significantly less positive staining than in controls (**Figures 10A, B**). Subsequently, RT-qPCR was performed to detect the expression levels of the pivotal genes. The results showed significant differences in mRNA expression levels of CRYBG1, RRM2, MMP1, and SLC19A2, with trends generally consistent with the bioinformatics analysis (**Figures 10C–F**). In addition, our one-way logistic regression analysis identified the independent predictive roles of these four hub genes in RA (**Figure 11A**). We then constructed a clinical nomogram model to measure the contribution of the characterized variables to RA prediction (**Figure 11B**). The ROC curves showed that the AUCs of both the nomogram and characterized variables were greater than 0.7, indicating good predictive ability (**Figure 11C**). Calibration, DCA, and CIC curves further supported the accuracy and clinical significance of the nomogram model (**Figures 11D–F**). Correlation analysis showed that CRYBG1 was significantly positively correlated with IgG; RRM2 was significantly positively correlated with ESR, ASO, RF, and C4; MMP1 was significantly positively correlated with NLR and ESR; and SLC19A2 was significantly negatively correlated with anti-CCP and C4 (**Figures 11G–P**). Overall, these results suggest that NET-related hub genes are strongly associated with clinical features of RA.

## 4 Discussion

RA is a common chronic inflammatory disease characterized by synovial thickening and inflammation, where abnormal immune cell infiltration plays a crucial role (33). Neutrophils play an intermediate role in the inflammatory cascade in RA (34). The formation of NETs, a programmed neutrophil death process, may be key to understanding inflammation initiation and perpetuation in the RA synovial microenvironment. In this study, we fully investigated key features of the RA synovial environment using scRNA-seq analysis and clarified the regulatory interactions network between neutrophils and other cells, especially fibroblasts. Second, we identified the most important hub genes closely related to NETs using multiple screening procedures. These genes were validated using external datasets, animal models, and clinical cohort samples. Most importantly, we identified the clinical relevance and immune infiltration landscape of hub genes. This comprehensive bioinformatics study and experimental validation provide valuable insights into RA pathogenesis and progression, paving the way for diagnostic model development and precision therapies.

Recent studies have characterized major cell types and corresponding transcriptomic features in the RA synovial microenvironment, identifying complex fibroblast subtypes and their communication networks with other cells (35). Interestingly, our analysis revealed seven major cellular compartments in the synovium: fibroblasts, macrophages, neutrophils, endothelial cells, monocytes, T cells, and B cells (**Figure 2C**). Neutrophils were the most abundant, suggesting that they play a key role in RA synovial pathology progression. Previous studies reported that neutrophils are the most abundant cells in the joint effusions and inflamed synovial tissues of patients with RA (36). Consistently, our work revealed predominant neutrophil infiltration in CIA rat joints, comprising approximately 57% of all synovial infiltrating immune cells (**Figures 2E, F**). FLS is an important structural effector cell in the synovium, and its tumor-like proliferation and invasiveness are typical pathological features of RA (37). Neutrophils promote the FLS inflammatory phenotype and upregulate major histocompatibility complex (MHC) class II through NET release, which is present in antigen-specific CD4+ T cells (38). Our intercellular communication analysis revealed significant interactions between neutrophils and fibroblasts, which is consistent with these findings (**Figure 3A**). We also found that neutrophil-fibroblast communication occurred mainly through the collagen, MIF, and Fn1 signaling pathways (**Figures 3B–D**). Notably, macrophage migration inhibitory factor (MIF) is a pleiotropic pro-inflammatory mediator that exerts a number of different cellular regulatory functions in RA. MIF not only promotes fibroblast proliferation and stimulates neutrophil chemotaxis and Th17 cell differentiation by increasing the expression of TLR4, but it also enhances RANKL-induced osteoclastogenesis thereby aggravating bone erosion in RA (39–41). In addition, MIF inhibitors have shown potent anti-inflammatory activity in macrophages and arthritis models (42). Some accumulating evidence found that MIF was able to promote the production of NETs by neutrophils through CXCR4, CXCR2 and stimulation of MAPK activation (43, 44). Our results also highlight the dominance of neutrophils in the CXCL signaling pathway that regulates immune response and inflammation (**Figure 3F**). A co-incubation experiment revealed a novel positive feedback loop involving NETs and FLS, whereby NETs stimulate FLS to secrete IL-33 and the chemokine CXCL8 via Toll-like receptors, which in turn exacerbates neutrophil recruitment and NETs production within the synovium (45). Taken together, these interesting findings enhance the understanding of RA synovial pathology, highlighting that interactions with fibroblasts and associated ligand pathways, centered on neutrophil infiltration, mediate inflammatory disturbances and pathological progression in the RA synovial microenvironment.

Next, we narrowed our observations from the synovial microenvironment to NETs, which are important strategies for neutrophils to execute their functions and have recently gained significant attention as a critical mechanism. By externalizing citrullinated autoantigens, NETs induce the production of anti-citrullinated peptide antibodies (ACPA), which further promote NET formation, an early event in RA that initiates autoimmune joint inflammation (46). Excessive NET release stimulates the production of cytokines like TNF-α, IL-6, IL-8, and IFN-γ, creating an “inflammatory storm” in patients with RA (47). In addition to the inflammatory phenotype, NETs can be internalized by FLS to enhance RANKL and matrix metalloproteinases production, which promotes osteoclast formation activation and exacerbates cartilage damage (48, 49). Immunofluorescence showed significant expression of MPO and NE — the two major products of reactive neutrophil infiltration and NETs — in patients with RA (**Figures 10A, B**). This study combined bulk RNA-seq data with multiple microarray datasets for differential gene and co-expression module identification to reveal genes associated with NETs. Bioconfidence enrichment analyses suggested that the pathogenesis of RA-NETs mainly focuses on positive regulation of protein phosphorylation, regulation of MAPK cascade and inflammatory regulatory pathways in the form of cytokine-cytokine receptor interactions and chemokine signaling pathways. The MAPK cascade, which consists of three key family members, ERK, JNK and p38, is involved in many cellular processes including cell growth, immunity, inflammation and stress response. Several studies have already documented that NETs-induced neutrophil activation occurs through pathways involving Akt, ERK1/2 and p38 phosphorylation (50, 51). Notably, several studies have emphasized the critical role of targeting NETs via MAPK signaling in RA therapy. Notably, growing evidence emphasizes the great potential of targeting NETs through MAPK signaling in RA therapy. For example, Yang et al. shared the report that lignans reduce NETs formation and inflammatory arthritis by inhibiting the Raf1-MEK-1-Erk axis (52). Overall, a comprehensive understanding of the upstream regulation of NETs could help to identify promising therapeutic targets and develop superior targeted therapeutics to treat RA.

Given the important role of NETs in RA, the development of early RA prediction models based on the association of key NETs can help us gain insight into and manage RA. We performed LASSO regression, SVE-RF and RF algorithms and crossed them, and ultimately chose the pivotal genes that were most prominently associated with NETs (CRYBG1, RRM2, MMP1, and SLC19A2) (**Figures 6I–N**). CRYBG1 (AIM1) is a common marker and therapeutic target in cancer. Initially, CRYBG1 was mainly localized to the putative chromosome 6 oncogene region of human melanoma and served as a good candidate suppressor because of its high expression level in suppressing melanoma cells (53). With the development of high-throughput technologies, CRYBG1 has been shown to be highly expressed in prostate cancer tissues (54). Although the role of CRYBG1 in different cancers is controversial, it is capable of interacting with the cytoskeleton. We identified CRYBG1’s potential role in RA management for the first time. RRM2, a key protein for DNA synthesis and repair, correlates significantly with DAS-28, compression pain, and swollen joints, with higher expression in remission RA (55). A study found that RRM2 promoted Akt phosphorylation and MMP9 expression, thereby promoting MH7A cell migration and invasion (56). Our findings showed that RRM2 was significantly and positively associated with ESR, ASO, RF, and C4 levels in patients with RA (**Figures 11H–L**). MMP1 is known to play an important role in the degradation and destruction of articular cartilage and bone and is closely associated with RA bone erosion (57). In a previous study, the inhibition of MMP1 expression reduced FLS migration and invasion, thereby linking RA inflammation and cartilage damage (58). SLC19A2 appears to be closely associated with megaloblastic anemia syndrome (59). We identified it for the first time as a key signature gene of RA-NETs and found that it was significantly associated with anti-CCP antibodies and C4 (**Figures 11O, P**). Of note, the expression of these pivotal genes was not only confirmed in our mapping of single cells, but also fully explored in our constructed AA rat synovial tissue and clinical cohorts. Using a nomogram diagnostic model, we mapped their unique value to identify the risk of RA (**Figures 11B–F**). Through a correlation analysis, we further emphasized their strong clinical relevance (**Figures 11G–P**). Immune infiltration analysis revealed an association with multiple immune cells involved in the RA immune microenvironment (**Figure 7I**). Interestingly, eosinophils showed a strong negative correlation with both MMP1 and RRM2, and a positive correlation with SLC19A2. Although we initially observed that they are differentially expressed in RA and show meaningful correlations with immune cells, further experiments are needed to verify their regulatory processes and immunological mechanisms in RA. In conclusion, understanding and exploring these genes is crucial for exploring novel diagnostic or prognostic biomarkers for RA and further searching for therapeutic targets.

In this study, we characterized and analyzed the intercellular communication of neutrophils in the RA synovium for the first time, providing a unique perspective for the molecular characterization of the RA synovial microenvironment. In addition, through the unprecedented integration of multiple datasets, we identified compelling diagnostic genes for RA-NETs. We constructed RA diagnostic models, based on which we further explored their immune landscapes and clinical relevance. Despite these important findings, this study has several limitations. First, the initially analyzed results were based on bioinformatic tools, and although confirmed at multiple levels, *ex vivo* and *in vivo*, sample-to-sample heterogeneity and differences should be explored. Second, due to the limited sample size and lack of prognostically relevant information, clinical trials with larger sample sizes and more diverse populations would help confirm the predictive models and improve the generalizability of the results. Despite our findings on the interactions between neutrophils and fibroblasts, further experiments are required to validate these phenomena. In particular, elucidating the processes and mechanisms by which these hub genes are involved in NET formation in the context of RA is crucial to characterize the therapeutic potential of NETs further. Presumably, further evidence and applications suggesting the relevance of NETs in RA pathogenesis and treatment will emerge.

## 5 Conclusion

In conclusion, our study emphasized the unique role of neutrophil-derived NETs in the RA synovial microenvironmental perspective. Through comprehensive bioinformatic analyses, we identified key hub genes associated with NETs, leading to the development of an accurate predictive RA diagnostic model and revealing a closely related immune infiltration landscape. These findings provide new insights into the inflammatory and immunological mechanisms that drive RA and suggest new directions for developing promising targeted diagnostic tools for RA.

## Data Availability

The datasets presented in this study can be found in online repositories. The names of the repository/repositories and accession number(s) can be found in the article/**Supplementary Material**.
