# Supplementary material for: Deciphering hub genes and immune landscapes related to neutrophil extracellular traps in rheumatoid arthritis: insights from integrated bioinformatics analyses and experiments
Source: Front Immunol. 2025 Jan 8;15:1521634. doi: 10.3389/fimmu.2024.1521634 (PMC11750673; doi:10.3389/fimmu.2024.1521634)
Supplement: Supplementary file 1 [file DataSheet1.docx]

Supplementary Table 1 Detailed information of the datasets used in this study

| GEO Series | Platform | Type | Organism | Source | Samples |
| --- | --- | --- | --- | --- | --- |
| GSE192504 | GPL21103 | scRNA-seq | Mice | Synovial cells | CIA mice (n=3) and healthy control mice (n=9) |
| [GSE5](https://www.ncbi.nlm.nih.gov/geo/query/acc.cgi?acc=GSE108112)5235 | GPL96 | microarray | Human | Synovial tisssue | RA (n=10), OA (n=10), and Normal (n=10) |
| [GSE5](https://www.ncbi.nlm.nih.gov/geo/query/acc.cgi?acc=GSE104948)5457 | GPL96 | microarray | Human | Synovial tisssue | RA (n=13), OA (n=10), and Normal (n=10) |
| GSE206848 | GPL570 | microarray | Human | Synovial tisssue | RA (n=2), OA (n=7), and Normal (n=7) |
| GSE150466 | GPL21290 | RNA-seq | Human | FLS | NETs (n=2) and non-NETs (n=2) |
| [GSE7](https://www.ncbi.nlm.nih.gov/geo/query/acc.cgi?acc=GSE104954)7298 | GPL570 | microarray | Human | Synovial tisssue | RA (n=16) and Normal (n=7) |

Supplementary Table 2 Primer sequences of RT-qPCR for genes

| Gene | Amplicon Size  （bp） | Forward primer  （5'→3'） | Reverse primer  （5'→3'） |
| --- | --- | --- | --- |
| CRYBG1 | 179 | CTGGGTGCTGGTTCTGCAAC | GGCATCGTCTTGTCCTCCTC |
| RRM2 | 140 | GGCTGGCTGTGACTTACCAT | AGCAGTGAGGCTGCATCTTT |
| MMP1 | 142 | GGGGCTTTGATGTACCCTAGC | TGTCACACGCTTTTGGGGTTT |
| SLC19A2 | 136 | GATCAGGCAGCGACCCTAGA | GAGTGAGGGTCAGGCACTTG |
| β-actin | 96 | CCCTGGAGAAGAGCTACGAG | GGAAGGAAGGCTGGAAGAGT |

Supplementary Table 3 Clinical characteristics of the population included in the study

| Variables | RA (n=30) | Normal (n=20) | Normal reference range | P value |
| --- | --- | --- | --- | --- |
| Gender (male/female) | 9 (30.00%)/21 (70.00%) | 8 (40.00%)/12 (60.00%) | NA | 0.465 |
| Age (year) | 55.50 (48.25, 67.00) | 55.00 (50.75, 62.75) | NA | 0.744 |
| NLR | 2.86 (1.96, 3.66) | NA | NA | NA |
| ESR (mm/h) | 25.00 (13.50, 55.25) | NA | 2-12 | NA |
| hs-CRP (mg/L) | 14.30 (5.53, 44.72) | NA | <5 | NA |
| IL-6 (pg/mL) | 14.26 (10.36, 37.71) | NA | 0-7 | NA |
| ASO (KIU/L) | 25.00 (17.75, 59.50) | NA | ≤200 | NA |
| RF (U/mL) | 109.35 (34.73, 293.10) | NA | ≤14 | NA |
| anti-CCP (U/mL) | 95.35 (21.13, 277.00) | NA | <4 | NA |
| IgA (g/L) | 2.32 (2.00, 3.16) | NA | 1-4.2 | NA |
| IgG (g/L) | 12.29 (10.62, 14.69) | NA | 8.6-17.4 | NA |
| IgM (g/L) | 1.42 (0.88, 2.22) | NA | 0.5-2.8 | NA |
| C3 (g/L) | 1.20 (1.10, 1.43) | NA | 0.7-1.4 | NA |
| C4 (g/L) | 0.25 (0.19, 0.28) | NA | 0.1-0.4 | NA |


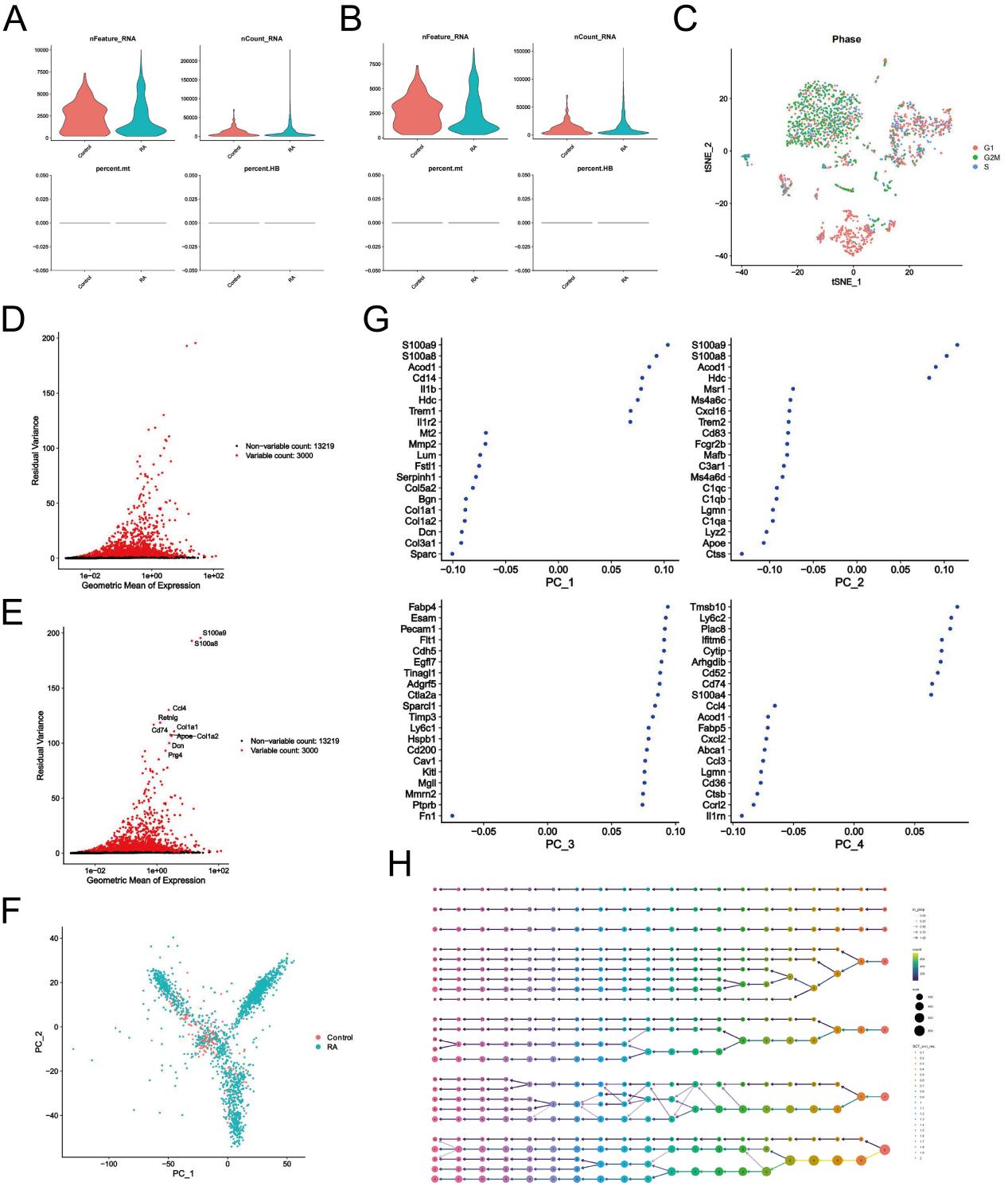


Supplementary Figure 1 Processing procedures for single-cell RNA analysis. (A-B) Quality control of the single-cell data. (C) Assessment of the cell-cycle score. (D-E) Find and display highly variable genes. (F) PCA was performed to reduce the data dimension. (G) Highly correlated genes for each PCA component. (H) clustree plot of the resolution parameters of dimensionality reduction clusters in single-cell data.
